# Impact of Omalizumab in Patients with Severe Uncontrolled Asthma and Possible Predictive Biomarkers of Response: A Real-Life Study

**DOI:** 10.3390/pharmaceutics15020523

**Published:** 2023-02-04

**Authors:** Susana Rojo-Tolosa, María Victoria González-Gutiérrez, José Antonio Sánchez-Martínez, Gonzalo Jiménez-Gálvez, Laura Elena Pineda-Lancheros, José María Gálvez-Navas, Alberto Jiménez-Morales, Cristina Pérez-Ramírez, Concepción Morales-García

**Affiliations:** 1 Respiratory Medicine Department, University Hospital Virgen de las Nieves, 18014 Granada, Spain; 2 Pharmacy Service, Pharmacogenetics Unit, University Hospital Virgen de las Nieves, 18014 Granada, Spain; 3 Center of Biomedical Research, Department of Biochemistry and Molecular Biology II, Institute of Nutrition and Food Technology “José Mataix”, University of Granada, Avda. del Conocimiento s/n., 18016 Granada, Spain

**Keywords:** severe uncontrolled asthma, omalizumab, effectiveness, biomarkers

## Abstract

Most patients with asthma can control their symptoms with a basic standard of medical care and with maintenance and rescue medication. However, between 5% and 10% of asthmatics worldwide do not achieve control of their symptoms and have recurrent exacerbations and respiratory difficulties. The objective of the study was the real-life evaluation of the clinical improvement of patients with severe eosinophilic asthma treated with omalizumab, together with the search for biomarkers associated with the response. An observational retrospective cohort study was conducted that included patients with severe uncontrolled allergic asthma being treated with omalizumab. Three types of response were evaluated: lower use of oral corticosteroids, improvement in lung function, and reduction in exacerbations. A total of 110 patients under treatment with omalizumab were included, with a mean age of 48 ± 16 years. After 12 months had elapsed, significant reductions were found in the number of exacerbations, use of oral cortico-steroids and doses of inhaled corticosteroids (*p* < 0.001). Lung function and asthma control improved significantly (*p* < 0.001; *p* = 0.004) and eosinophil levels were significantly reduced (*p* = 0.004). Low scores in the Asthma Control Test were associated with the oral corticosteroid-saving effect; lower previous FEV1 levels and absence of chronic obstructive pulmonary disease (COPD) were related to improvement in lung function, and prior FEV1 values higher than 80% and absence of gastroesophageal reflux disease (GERD) with a reduction in exacerbations. The results of this study confirm the clinical benefit obtained after the introduction of omalizumab and the possible predictive biomarkers of response to the treatment.

## 1. Introduction

Asthma is a chronic respiratory disease with a high incidence worldwide, characterized by a chronic obstruction of the airways, which causes patterns of inflammation and bronchial remodeling [1,2,3]. Most patients with asthma can control their symptoms with a basic standard that includes control and maintenance medication, administered over prolonged periods including inhaled corticosteroids (ICS), leukotriene receptor antagonists and long-acting β2 adrenergic agonists, and rescue medication, used on demand to provide fast relief or prevent bronchoconstriction [4,5,6,7]. However, despite adaptation of standard therapy to the patient’s needs, between 5% and 10% of asthmatics worldwide do not achieve control of their symptoms and suffer recurrent exacerbations and a lack of control of respiratory symptoms [8,9]. The Spanish Asthma Management Guidelines (GEMA) and the Global Initiative for Asthma (GINA) consider these patients as cases of severe uncontrolled asthma (SUA) and according to their treatment indications, GEMA step 6 and GINA step 5, the introduction of biological therapies suited to the patient’s phenotype/endotype is advised to increase overall control of the disease [6,7].

Immunoglobulin E (IgE) is a type of antibody related to hypersensitivity and allergic reactions, which causes an immune response by binding to the high-affinity receptor FcεRI located on the surface of mast cells and basophils, promoting the release of inflammatory mediators [10].

Omalizumab (Xolair^®^) is a humanized monoclonal antibody that binds selectively to IgE and prevents it from binding to the FcεRI receptor in basophils and mast cells, thereby reducing the amount of free IgE available to trigger the allergic cascade [11,12]. By blocking IgE from binding to FcεRI receptors, omalizumab achieves a 97% reduction in the expression of these receptors on the surface of mast cells and basophils [13]. Moreover, because of the binding of omalizumab to free IgE, blood IgE levels decrease by between 96% and 99%, and as a result of the depletion of free IgE, the plasma membrane FcεRI receptors of basophils are transferred to the cytoplasm and are not resynthesized [14,15].

Although multiple randomized controlled clinical trials have been conducted that have demonstrated the efficacy and safety of omalizumab [16,17,18,19,20,21,22,23,24,25,26,27,28,29,30], they do not reflect the situations that arise in common clinical practice as they were developed in the optimum conditions with strict inclusion and exclusion criteria. Consequently, real-life studies have become increasingly important to support the results reported by clinical trials. There are now real-life studies demonstrating the efficacy of omalizumab [31,32,33,34,35,36]. These studies include diverse groups of participants, so accurate pooled estimates are needed to assess the efficacy and determine the biomarker predictors of response in specific populations. The aim of this study was to evaluate the effectiveness of omalizumab treatment and identify predictive markers of response in Caucasian patients in southern Spain.

## 2. Materials and Methods

We conducted a real-life observational retrospective cohort study.

### 2.1. Study Population

This study included 113 patients aged over 18 years and of Caucasian origin diagnosed with SUA according to GEMA 5.1 criteria, recruited in the Respiratory Medicine Department of the Hospital Universitario Virgen de las Nieves de Granada (Spain) between March 2007 and April 2022. Out of the 113 patients recruited, the response was evaluated in 110 patients treated with omalizumab, prior to beginning treatment and when 12 months had elapsed from the start of the biological therapy. The administration route of the drug was subcutaneous, with doses of 75 mg to 600 mg, depending on the initial IgE concentration and the patient’s weight, every 2 or 4 weeks [37]. The remaining patients did not meet the study’s evaluation criteria.

### 2.2. Socio-Demographic and Clinical Variables

The socio-demographic variables included age, sex, body mass index (BMI), smoking status, years with the disease, nasal polyps, previous respiratory disease, allergies, gastroesophageal reflux disease (GERD), sleep apnea-hypopnea syndrome (SAHS), chronic obstructive pulmonary disease (COPD), years with omalizumab, treatment dose, and change to another monoclonal antibody therapy.

The clinical variables were collected according to the 12 months prior to starting omalizumab treatment and after completing the first year of treatment. They included oral corticosteroid (OCS) and ICS doses, blood eosinophil count, exacerbations requiring emergency treatment and/or hospitalization, IgE, lung function as percentage forced expiratory volume in the first second (%FEV1), and Asthma Control Test (ACT) [38].

### 2.3. Statistical Analysis

The quantitative variables were expressed as the mean (±standard deviation) for those that complied with normality and as the median and percentiles (25 and 75) for those that did not follow a normal distribution. Normality was confirmed using the Kolmogorov–Smirnov test.

The clinical variables responsible for the response were compared before and after treatment using the McNemar test for qualitative variables. For quantitative variables that complied with normality, we used the t test for paired data and the Mann–Whitney U test (Wilcoxon rank sum test) for non-normal variables. The results were considered significant when the *p* value was less than 0.05.

To evaluate the predictors of response at 12 months, the following were taken as response variables: reduction in OCS cycles per year, considering a reduction of at least 50% in the cycles or absence of OCS as a satisfactory response; improvement in lung function, considering those that achieved an increase of at least 10% in FEV1 after 12 months of treatment as responsive; and reduction in exacerbations per year requiring emergency treatment and/or hospitalization, taking a reduction of at least 50% or absence of exacerbations as a satisfactory response. The bivariate analysis between the response and socio-demographic and clinical variables was performed using Pearson’s chi-squared test or applying Fisher’s exact test for the qualitative variables. For the quantitative variables, the Student’s *t*-test was applied to the variables that complied with normality. The Mann–Whitney U test was applied for non-normal variables. A multivariate (logistic or linear regression) analysis was used to calculate the adjusted odds ratio (OR) and the 95% confidence interval (CI) for the possible prognostic factors of response to OCS, lung function, and exacerbations. All of the tests were 2-sided, with a probability of 0.05 or less considered statistically significant, and were performed with the R 4.2.0 free software.

## 3. Results

### 3.1. Characteristics of the Patients Treated with Omalizumab

The clinical and demographic characteristics of the 110 patients treated with omalizumab are described in Table 1. In total, 61.82% of the patients were women (68/110); their mean age was 48.48 ± 16.08 years, with a median of 8.5 [5–13.75] years with the disease and 2.5 [1–5] years under treatment with omalizumab. Most of the patients were overweight or obese, 40% (44/110) and 34.55% (38/110), respectively, and 4.55% were smokers (5/110). There were 20.91% (23/110) who had had some previous respiratory disease, 19.09% (21/110) with nasal polyps, 75.45% (83/110) with allergies, 27.27% (30/110) with GERD, 18.18% (20/110) with SAHS, and 14.55% (16/110) with COPD.

All of the patients had required ICS in the year before beginning the treatment, with a median of 320 [184–640] mg/day, and 77.27% (85/110) needed OCS, with a median of two [1–3] cycles. Mean %FEV1 was 76.12 ± 21.68, and a median blood eosinophil count of 250 [127.5–485] cells/μL was recorded, and IgE of 310.45 [142.5–716.25] IU/mL. There were 61.82% (68/110) who suffered at least one exacerbation during the year prior to the treatment and the median ACT score was 12 [10–16] points. Of the 110 patients, 42.73% required changing to another monoclonal therapy for asthma control.

### 3.2. Clinical Effectiveness of Omalizumab

The effectiveness of omalizumab was assessed in 110 (97.35%) patients of the 113 candidates for the study (Table 2). The treatment was suspended in 2.65% of the patients (3/113) due to the presence of adverse effects.

After a year of treatment with omalizumab, the 50% reduction in OCS was satisfactory in 51.38% (56/110) of the patients and 14.68% (16/110) did not require OCS during the first 12 months of biological therapy. The %FEV1 increased by at least 10% in 46.15% (42/91) of cases and 75.53% (71/94) showed FEV1 values higher than 80%. The reduction in at least 50% in exacerbations requiring emergency department treatment and/or hospitalization was satisfactory in 49.09% (54/110) of the patients and 34.55% (38/110) had no exacerbations.

### 3.3. Comparison of Clinical Variables before and after Treatment

#### 3.3.1. Daily Dose of ICS

After 12 months of biological therapy with omalizumab, daily doses of ICS showed significant reductions of 42.50% (*p* < 0.001). The results of the comparative analysis are shown in Table 3.

#### 3.3.2. OCS Cycles per Year

**Figure 1:**
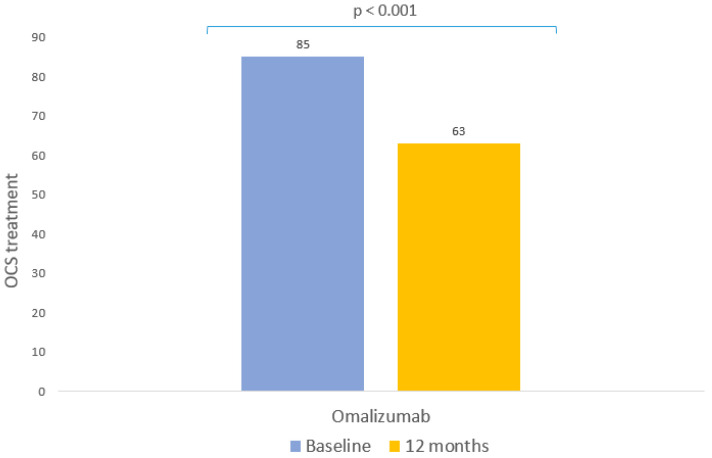
Clinical response to omalizumab: OCS. Patients with oral corticosteroid requirements during the 12 months prior to starting omalizumab and at the follow-up evaluation after 12 months of therapy.

During the year prior to starting biological therapy, a high use of OCS was recorded among the patients in the study: 77.27% of them needed at least one cycle of OCS. After a year with omalizumab, the use of OCS had fallen significantly, by 25.88% (*p* < 0.001, Table 3, Figure 1) and in parallel with this, a significant reduction occurred in the median doses of OCS recorded (*p* < 0.001, Table 3, Figure 1).

#### 3.3.3. Lung Function

**Figure 2:**
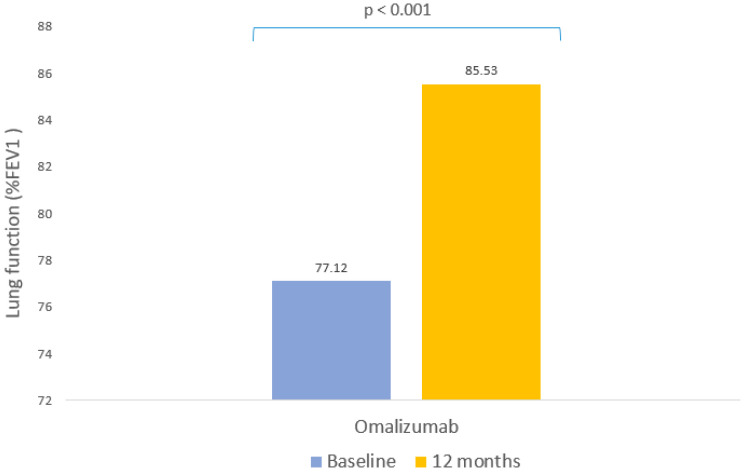
Clinical response to omalizumab: lung function. Mean percentage forced expiratory volume in the first second (%FEV1) before omalizumab and after 12 months of treatment.

Lung function improved significantly after 12 months with omalizumab, showing an increase of 9.41% in the mean FEV1 (*p* < 0.001, Table 3, Figure 2). However, there were no statistically significant differences in the group of patients with FEV1 values greater than 80%.

#### 3.3.4. Asthma Control Test (ACT)

After the administration of omalizumab, patients had a significant increase of 8.5 in the median ACT score (12 to 20.5; *p* < 0.004, Table 3).

#### 3.3.5. Frequency of Severe Exacerbations

**Figure 3:**
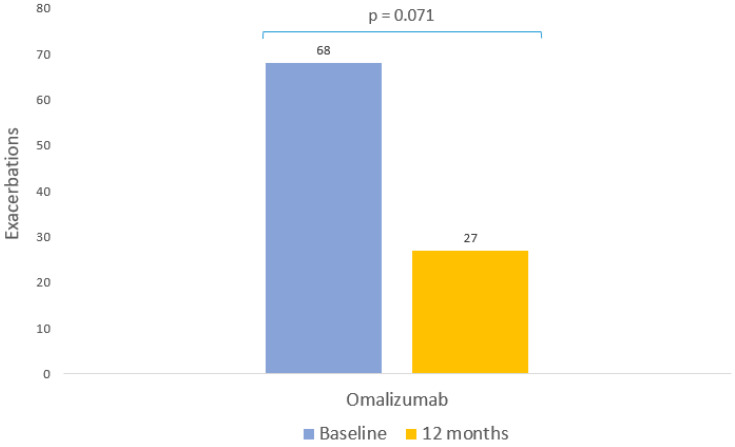
Clinical response to omalizumab: exacerbations. Patients with exacerbations in the 12 months prior to starting omalizumab and after 12 months of therapy.

The percentage of patients with exacerbations decreased from 61.82% to 24.55% after a year of omalizumab treatment (*p* = 0.071, Table 3, Figure 3); the median number of exacerbations also decreased (*p* < 0.001, Table 3).

#### 3.3.6. Inflammatory Markers

**Figure 4:**
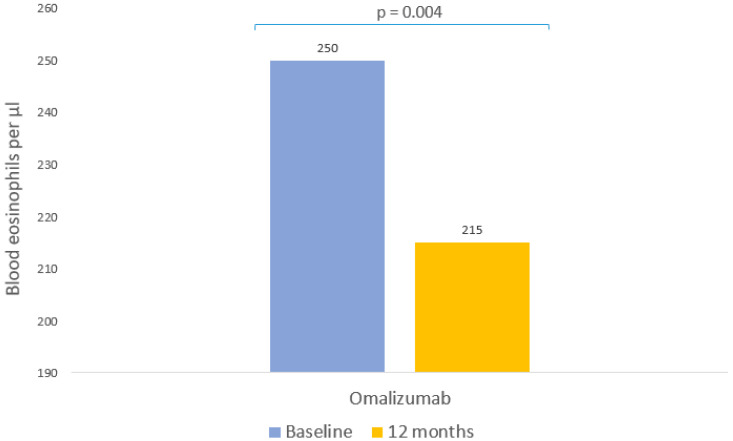
Clinical response to omalizumab: blood eosinophils. Median eosinophils prior to omalizumab and after 12 months of treatment.

Omalizumab was associated with a significant reduction of 14% in the blood eosinophil counts (*p* = 0.004, Table 3, Figure 4).

#### 3.3.7. Immunoglobulin E

No statistically significant changes in IgE levels were found after 12 months of treatment with omalizumab (*p* = 0.888, Table 3).

### 3.4. Predictors of Response at 12 Months

#### 3.4.1. Response to Reduction in Oral Corticosteroids (OCS)

In the bivariate analysis, a greater response to OCS was found in patients with lower baseline ACT values, higher blood eosinophil levels, and lower treatment doses every 4 weeks (Table S1). The multivariate analysis showed that the independent variable associated with OCS response at 12 months was lower at baseline ACT values (OR = 0.74; 95% CI = 0.53–0.97). The results of the multivariate analysis are shown in Table 4.

The multivariate analysis was adjusted by ACT, eosinophil levels, and omalizumab dose for predictors of OCS reduction; sex, COPD, and previous FEV1 value for predictors of lung improvement; polyps and GERD for predictors of exacerbation reduction.

#### 3.4.2. Lung Function Response (FEV1)

In the bivariate analysis, satisfactory lung function response was associated with women, absence of COPD linked to asthma, and lower prior FEV1 values (the values are shown in detail in Table S2). After the multivariate analysis was performed, we found that lower initial FEV1 values (OR = 0.93; 95% CI = 0.90–0.96; Table 4) and absence of COPD (OR = 34.26; 95% CI = 5.20–395.88; Table 4) indicated greater improvement in lung function.

#### 3.4.3. Response to Reduction of Exacerbations

The bivariate analysis associated the absence of polyps and GERD and higher previous FEV1 values (the values are shown in detail in Table S3). In the multivariate analysis, a significant association with response was found in the absence of GERD (OR = 3.08; 95% CI = 1.09–8.77; Table 4) and higher previous FEV1 values (OR = 3.24; 95% CI = 1.06–12.23; Table 4).

## 4. Discussion

In this study, omalizumab was associated with a significant improvement in lung function (FEV1) and the control of asthma symptoms, evaluated using the ACT questionnaire, and significant reductions in the rate of severe exacerbations per year, blood eosinophil level, OCS cycles per year, and ICS doses. The response to treatment with omalizumab achieved in our study is in line with the previous published real-life studies, collected by Jean Bousquet et al. in their meta-analysis published in 2021, which found significant improvements in lung function (95% CI: 0.03–0.48; *p* = 0.02), annual exacerbation rate (risk ratio [RR]: 0.41; 95% CI: 0.30–0.56; *p* < 0.01), ACT questionnaire score (mean difference [MD]: 6.47; 95% CI: 4.76–8.18), and in the proportion of patients that received OCS, which fell significantly (RR: 0.59; 95% CI: 0.47–0.75; *p* < 0.01) [33]. It should be noted that we found no statistically significant differences in the group of patients with FEV1 values over 80% after 12 months of treatment with omalizumab, which may indicate that this biological therapy achieves improvements in lung function, but in many cases, with very low baseline FEV1 values, a very significant increase is not achieved in the first year of biological therapy. As for biomarkers, the multivariate analysis indicated that having a positive response of reduction in OCS was more likely if the subject had lower initial ACT values. As lung function response markers, it was found that the absence of COPD and lower prior %FEV1 values led to a better response, and finally, the absence of GERD and higher prior %FEV1 values were the markers associated with a positive response of reduction in exacerbations. The previous literature showed different results. Casale et al. associated improved lung function in those patients with a high level of eosinophils (*p* = 0.011) and a higher risk of exacerbations with omalizumab if they had been suffered the previous year (OR: 2.19; 95% CI: 1.55–3.08; *p* < 0.001), which is in line with our study, and they did not look for biomarkers associated with the OCS-saving effect [34]. Other authors have associated biomarkers with overall response and considered lower FEV1 levels and a higher eosinophil count or higher IgE levels as predictors of response to omalizumab [35,36,37,38].

This study, being a real-life investigation, lacked a placebo control group, and this could be considered its main limitation. The absence of a control group means that the magnitude of the results observed lacks the firmness of a comparison with a control group. Another inherent limitation of retrospective studies is the lack of data collection such as in the ACT and the sample size. Nevertheless, although randomized clinical trials remain the gold standard, this type of study makes it possible to extrapolate these results to uncontrolled and heterogeneous settings.

## 5. Conclusions

Omalizumab was the first biological therapy approved for the treatment of asthma and has brought about a great improvement in the quality of life of patients with severe allergic asthma. Its efficacy and safety have been demonstrated in numerous controlled clinical trials and the results attained in this study show very promising data in real life. Omalizumab achieved significant improvements in the three responses evaluated, reducing the use of oral corticosteroids, improving lung function, and decreasing and/or preventing the presence of exacerbations in many of the patients studied. In addition, response markers could be a useful tool for making decisions in clinical practice.

## Figures and Tables

**Table 1:** Demographic and clinical characteristics of patients treated with omalizumab.

	N	%	Mean ± SD/p_50_ (p_25_, p_75_)
Age	110		48.48 ± 16.08
Sex			
Women	68	61.82	
Men	42	38.18	
BMI			
Underweight	1	0.91	
Normal weight	27	24.55	
Overweight	44	40	
Obesity	38	34.55	
Tobacco consumption			
Non-smoker	77	70	
Former smoker	28	25.45	
Current smoker	5	4.55	
Previous respiratory disease			
Yes	23	20.91	
No	87	79.09	
Polyps			
Yes	21	19.09	
No	89	80.91	
Allergies			
Yes	83	75.45	
No	27	24.55	
GERD			
Yes	30	27.27	
No	80	72.73	
SAHS			
Yes	20	18.18	
No	90	81.82	
COPD			
Yes	16	14.55	
No	94	85.45	
Years with EA	110		8.5 [5–13.75]
ICS (mg/day)	110		320 [184–640]
OCS cycles per year	110		2 [1–3]
Yes	85	77.27	
No	25	22.73	
Baseline %FEV1	106		76.12 ± 21.68
<80	62	58.49	-
>80	44	41.51	-
Baseline ACT	25		12 [10–16]
Exacerbation in previous year	110		1 [0–2]
Yes	68	61.82	
No	42	38.18	
Baseline blood eosinophil count (cells/μL)	100		250 [127.5–485]
Baseline IgE (IU/mL)	96		310.45 [142.5–716.25]
Years with omalizumab	110		2.5 [1–5]
Omalizumab dose (mg/4 weeks)	110		450 [300–600]
Change of BT			
Yes	47	42.73	
No	63	57.27	

BMI: body mass index; GERD: gastroesophageal reflux disease; SAHS: sleep apnea-hypopnea syndrome; COPD: chronic obstructive pulmonary disease; EA: eosinophilic asthma; ICS: inhaled corticosteroids; OCS: oral corticosteroids; %FEV1: percentage forced expiratory volume in the first second; ACT: Asthma Control Test; IgE: immunoglobulin E; BT: biological therapy. Qualitative variables are shown as numbers (percentage, %). Quantitative variables with normal distribution are shown as the mean ± standard deviation (SD). Quantitative variables with non-normal distribution are shown as p50 (p25–p75).

**Table 2:** Clinical efficacy of omalizumab in patients with severe uncontrolled asthma.

Response Definition	N	%
OCS reduction ≥ 50%		
Yes	56	50.91
No	37	33.63
No OCS	17	15.45
OCS reduction ≥ 50% or absence		
Yes	71	64.55
No	39	35.45
FEV1 increase ≥ 10%		
Yes	42	46.15
No	49	53.85
FEV1 increase ≥ 10% or FEV1 ≥ 80%		
Yes	71	75.53
No	23	24.47
Exacerbation reduction ≥ 50%		
Yes	54	49.09
No	18	16.36
No exacerbations	38	34.55
Exacerbation reduction ≥ 50% or absence		
Yes	90	81.82
No	20	18.18

OCS: oral corticosteroids; FEV1: maximum forced expiratory volume in the first second.

**Table 3:** Changes in the baseline variables after 12 months of biological therapy.

Independent Variable	Biological Therapy with Omalizumab
ICS dose (mg/day)	
Baseline, p50 (p25, p75)	320 [184–640]
Follow-up, p50 (p25, p75)	184 [92.75–400]
Change from baseline	*p* < 0.001
Use of OCS (Yes/No)	
Baseline, n (%)	85 (77.27)
Follow-up, n (%)	63 (57.27)
Change from baseline	*p* < 0.001
Cycles of OCS per year	
Baseline, p50 (p25, p75)	2 [1–3]
Follow-up, p50 (p25, p75)	1 [0–2]
Change from baseline	*p* < 0.001
Patients with FEV1 >80%	
Baseline, n (%)	44 (41.51)
Follow-up, n (%)	53 (56.38)
Change from baseline	*p* = 0.745
%FEV1	
Baseline, mean (SD)	76.12 ± 21.68
Follow-up (SD)	85.53 ± 20.10
Change from baseline	*p* < 0.001
ACT	
Baseline, p_50_ (p_25_, p_75_)	12 [10–16]
Follow-up, p_50_ (p_25_, p_75_)	20.5 [16–23]
Change from baseline	*p* = 0.004
Presence of exacerbations (Yes/No)	
Baseline, n (%)	68 (61.82)
Follow-up, n (%)	27 (24.55)
Change from baseline	*p* = 0.071
Exacerbations per year	
Baseline, p_50_ (p_25_, p_75_)	1 [0–2]
Follow-up, p_50_ (p_25_, p_75_)	0 [0–0]
Change from baseline	*p* < 0.001
Blood eosinophils (cell/μL)	
Baseline, p_50_ (p_25_, p_75_)	250 [127.5–485]
Follow-up, p_50_ (p_25_, p_75_)	215 [120–327.5]
Change from baseline	*p* = 0.004
IgE (IU/mL)	
Baseline, p_50_ (p_25_, p_75_)	310.45 [142.5–716.25]
Follow-up, p_50_ (p_25_, p_75_)	496 [191–852]
Change from baseline	*p* = 0.888

ICS: inhaled corticosteroids; OCS: oral corticosteroids; %FEV1: percentage forced maximum expiratory volume in the first second; ACT: Asthma Control Test; IgE: immunoglobulin E. Qualitative variables are shown as numbers (percentage, %). Quantitative variables with normal distribution are shown as the mean ± standard deviation (SD). Quantitative variables with a non-normal distribution are shown as p50 (p25–p75).

**Table 4:** Response predictors after 12 months of treatment with omalizumab in patients with severe uncontrolled asthma (multivariate analysis).

	B	Odds Ratio	*p*-Value	95% CI
OCS reduction predictors				
ACT	−0.2994	0.74	0.045	0.53–0.97
Lung improvement predictors				
FEV1	−0.0719	0.93	<0.001	0.90–0.96
COPD	3.5341	34.26	0.001	5.20–395.88
Exacerbation reduction predictors				
GERD	1.1238	3.08	0.033	0.11–0.92
FEV1 (>80)	1.1759	3.24	0.054	1.06–12.23

OCS: oral corticosteroids; FEV1: maximum forced expiratory volume in the first second; ACT: Asthma Control Test; COPD: chronic obstructive pulmonary disease; GERD: gastroesophageal reflux disease. CI: confidence interval.

## Data Availability

Not applicable.

## Supplementary Materials

The following supporting information can be downloaded at: https://www.mdpi.com/article/10.3390/pharmaceutics15020523/s1, Table S1: Predictors of oral corticosteroid reduction at 12 months of omalizumab treatment in patients with severe uncontrolled asthma (bivariate analysis); Table S2: Predictors of lung function improvement at 12 months of omalizumab treatment in patients with severe uncontrolled asthma (bivariate analysis); Table S3: Predictors of exacerbation reduction at 12 months of omalizumab treatment in patients with severe uncontrolled asthma (bivariate analysis).
