# Supplementary material for: Impact of Omalizumab in Patients with Severe Uncontrolled Asthma and Possible Predictive Biomarkers of Response: A Real-Life Study
Source: Pharmaceutics. 2023 Feb 4;15(2):523. doi: 10.3390/pharmaceutics15020523 (PMC9962091; doi:10.3390/pharmaceutics15020523)
Supplement: Supplementary file 1 [file pharmaceutics-15-00523-s001.zip › pharmaceutics-2106075-supplementary.pdf]

Supplementary Materials: Impact of Omalizumab in Patients with Severe Uncontrolled Asthma and Possible Predictive Biomarkers of Response: A Real-Life Study

Susana Rojo-Tolosa, María Victoria González-Gutiérrez, José Antonio Sánchez-Martínez, Gonzalo Jiménez-Gálvez, Laura Elena Pineda-Lancheros, José María Gálvez-Navas, Alberto Jiménez-Morales, Cristina Pérez-Ramírez and Concepción Morales-García

Table S1. Predictors of oral corticosteroid reduction at 12 months of omalizumab treatment in patients with severe uncontrolled asthma (bivariate analysis).

	**Response to oral corticosteroid reduction**						
Independent variable	N	Unsatisfactory	Successful	p-value	Reference category	OR	$CI_{95\%}$
Age	110	51.62 ± 2.76	46.86 ± 15.55	0.132	-	-	-
Sex							
Female	68	23 (33.8)	45 (66.2)	0.649	-	-	-
Male	42	16 (38.1)	26 (61.9)				
BMI							
Underweight	1	1 (100)	0 (0)	0.086*	-	-	-
Normal weight	27	5 (18.5)	22 (81.5)				
Overweight	44	18 (40.9)	26 (59.1)				
Obesity	38	15 (39.5)	23 (60.5)				
Tobacco consumption							
Non smoker	77	29 (37.7)	48 (62.3)	0.674	-	-	-
Former smoker	28	8 (28.6)	20 (71.4)				
Current smoker	5	2 (40.0)	3 (60.0)				
Previous respiratory disease							
Yes	23	10 (43.5)	13 (56.5)	0.366	-	-	-
No	87	29 (33.3)	58 (66.7)				
Polyps							
Yes	21	10 (47.6)	11 (52.4)	0.195	-	-	-
No	89	29 (32.6)	60 (67.4)				
Allergies							
Yes	83	30 (36.1)	53 (63.9)	0.791	-	-	-
No	27	9 (33.3)	18 (66.7)				
GERD							
Yes	30	14 (46.7)	16 (53.3)	0.132	-	-	-

No	80	25 (31.2)	55 (68.8)				
SAHS							
Yes	20	10 (50)	10 (50)	0.133	-	-	-
No	90	29 (32.2)	61 (67.8)				
COPD							
Yes	16	6 (37.5)	10 (62.4)	0.853	-	-	-
No	94	33 (35.1)	61 (64.9)				
Years with AE	110	10 [5-14]	8 [4-13.5]	0.651	-	-	-
ICS (mg/day)	110	320 [184-620]	400 [184-640]	0.472	-	-	-
OCS cicles per year	110	2 [0.5-3]	2 [1-3]	0.788	-	-	-
Yes	85	29 (34.1)	56 (65.9)	0.589	-	-	-
No	25	10 (40)	15 (60)				
Baseline FEV1 (%)	106	72.74 ± 21.25	77.96 ± 21.86	0.245	-	-	-
Baseline ACT	25	15 [12.5-16.5]	11 [9-13]	0.045	-	0.74	[0.53-0.97]
Exacerbation in previous year	110	1 [0-2]	1 [0-2]	0.807	-	-	-
Yes	68	26 (38.2)	42 (61.8)	0.438	-	-	-
No	42	13 (31)	29 (69)				
Basal blood eosinophils (cell/mcl)	100	195 [110.3-347.5]	300 [152.5-550]	0.067	-	-	-
Baseline IgE (IU/Ml)	96	319.5 [142.5-666]	310.5 [143-752.9]	0.910	-		
Years with omalizumab	110	3 [1-5]	2 [1-5]	0.746	-		
Omalizumab dose (mg/4 weeks)	110	600 [300-750]	300 [150-450]	0.052	-	0.99	[0.99-1]

BMI, body mass index; GERD, gastro-oesophageal reflux disease; SAHS, sleep apnoea-hypopnoea syndrome; COPD, chronic obstructive pulmonary disease; EC, eosinophilic asthma; ICS, inhaled corticosteroids; OCS, oral corticosteroids; FEV1, peak expiratory volume in the first second of forced expiration; ACT, Asthma Control Test; IgE, immunoglobulin E; OR, Odds ratio; CI95%, 95% confidence interval.

*Fisher's exact test

Table S2. Predictors of lung function improvement at 12 months of omalizumab treatment in patients with severe uncontrolled asthma (bivariate analysis).

	**Response to improved lung function**						
Independent variable	N	Unsatisfactory	Satisfactory	p-value	Reference category	OR	$CI_{95\%}$
Age	91	48.16 ± 15.66	49.73 ± 16.48	0.638	-	-	-
Sex							
Female	57	26 (45.6)	31 (54.4)	0.041	Male	2.49	[1.04-6.22]
Male	34	23 (67.6)	11 (32.4)				

BMI							
Underweight	-	-	-	1*	-	-	-
Normal weight	23	12 (52.2)	11 (47.8)				
Overweight	30	16 (53.3)	14 (46.7)				
Obesity	38	21 (55.3)	17 (44.7)				
Tobacco consumption							
Non smoker	5	2 (40)	3 (60)	0.656*	-	-	-
Former smoker	21	10 (47.6)	11 (52.4)				
Current smoker	65	37 (56.9)	28 (43.1)				
Previous respiratory disease							
Yes	20	11 (55)	9 (45)	0.907	-	-	-
No	71	38 (53.5)	33 (46.5)				
Polyps							
Yes	18	8 (44.4)	10 (55.6)	0.372	-	-	-
No	73	41 (56.2)	32 (43.8)				
Allergies							
Yes	66	33 (50)	33 (50)	0.232	-	-	-
No	25	16 (64)	9 (36)				
GERD							
Yes	24	13 (54.2)	11 (45.8)	0.971	-	-	-
No	67	36 (53.7)	31 (46.3)				
SAHS							
Yes	18	12 (66.7)	6 (33.3)	0.223	-	-	-
No	73	37 (50.7)	36 (493)				
COPD							
Yes	12	10 (83.3)	2 (16.7)	0.029	Yes	5.13	[1.25-34.77]
No	79	39 (49.4)	40 (50.6)				
Years with AE	91	9 [5-15]	9 [7-12]	0.913	-	-	-
ICS (mg/day)	91	400 [184-640]	440 [200-640]	0.398	-	-	-
OCS cicles per year	91	2 [0-3]	2 [1-3]	0.603	-	-	-
Yes	69	35 (50.7)	34 (49.3)	0.2901	-	-	-
No	22	14 (63.6)	8 (36.4)				
Baseline FEV1 (%)	91	82.73 ± 19.95	65.48 ± 18.15	<0.001	-	0.95	[0.92-0.98]
<80	55	22 (40)	33 (60)	0.001	>80	4.50	[1.83-11.86]
>80	36	27 (75)	9 (25)				
Baseline ACT	76	10 [9.5-14]	11.5 [10.8-15.3]	0.531	-	-	-
Exacerbation in previous year	91	1 [0-2]	1 [0-2]	0.711	-	-	-

Yes	57	31 (54.4)	26 (45.6)	0.894	-	-	-
No	34	18 (52.9)	16 (47.1)				
Basal blood eosinophils (cell/mcl)	81	290 [145-465]	255 [160-567.5]	0.534	-	-	-
Baseline IgE (IU/Ml)	80	415.5 [145.5-1012.5]	342 [158.5-689.3]	0.685	-	-	-
Years with omalizumab	91	3 [1-5]	4 [1.3-5.8]	0.368	-	-	-

BMI, body mass index; GERD, gastro-oesophageal reflux disease; SAHS, sleep apnoea-hypopnoea syndrome; COPD, chronic obstructive pulmonary disease; EC, eosinophilic asthma; ICS, inhaled corticosteroids; OCS, oral corticosteroids; FEV1, peak expiratory volume in the first second of forced expiration; ACT, Asthma Control Test; IgE, immunoglobulin E; OR, Odds ratio; CI95%, 95% confidence interval.

Table S3. Predictors of exacerbation reduction at 12 months of omalizumab treatment in patients with severe uncontrolled asthma (bivariate analysis).

	**Response to exacerbation reduction**						
Independent variable	N	Unsatisfactory	Satisfactory	p-value	Reference category	OR	$CI_{95\%}$
Age	110	50.65 ± 12.58	48 ± 16.78	0.504	-	-	-
Sex							
Female	68	12 (17.6)	56 (82.4)	0.853	-	-	-
Male	42	8 (19)	34 (81)				
BMI							
Underweight	1	0 (0)	1 (100)	0.076*	-	2.17e-06	[NA-5.72e+122]
Normal weight	27	2 (7.4)	25 (92.6)				
Overweight	38	5 (13.2)	33 (86.8)				
Obesity	44	13 (29.5)	31 (70.5)				
Tobacco consumption							
Non smoker	77	15 (19.5)	62 (80.5)	0.906*	-	-	-
Former smoker	28	4 (14.3)	24 (85.7)				
Current smoker	5	1 (20)	4 (80)				
Previous respiratory disease							
Yes	23	4 (17.4)	19 (82.6)	0.912	-	-	-
No	87	16 (18.4)	71 (81.6)				

Polyps							
Yes	21	7 (33.3)	14 (66.7)	0.045	Yes	2.93	[0.96-8.54]
No	89	13 (14.6)	76 (85.4)				
Allergies							
Yes	83	17 (20.5)	66 (79.5)	0.273	-	-	-
No	27	3 (11.1)	24 (88.9)				
GERD							
Yes	30	10 (33.3)	20 (66.7)	0.012	Yes	3.50	[1.27-9.73]
No	80	10 (12.5)	70 (87.5)				
SAHS							
Yes	20	6 (30)	14 (70)	0.196	-	-	-
No	90	14 (15.6)	76 (84.4)				
COPD							
Yes	16	3 (18.8)	13 (81.2)	0.949	-	-	-
No	94	17 (18.1)	77 (81.9)				
Years with AE	110	9 [5-12]	8 [4.3-14]	0.810	-	-	-
ICS (mg/day)	110	400 [184-850]	320 [184-640]	0.302	-	-	-
OCS cicles per year	110	2 [0-4]	2 [1-3]	0.735	-	-	-
Yes	85	14 (16.5)	71 (83.5)	0.391		-	-
No	25	6 (24)	19 (76)				
Baseline FEV1 (%)	106	68 ± 18.25	78.01 ± 22.07	0.067	-	1.02	[0.99-1.04]
<80	62	16 (25.8)	46 (74.2)	0.030	<80	3.48	[1.16-12.90]
>80	44	4 (9.1)	40 (90.9)				
Baseline ACT	25	13.5 [10.8-16]	12 [9-15]	0.551	-	-	-
Exacerbation in previous year	110	1 [0-2]	1 [0-2]	0.756	-	-	-
Yes	68	14 (20.6)	54 (79.4)	0.405	-	-	-
No	42	6 (14.3)	36 (85.7)				
Basal blood eosinophils (cell/mcl)	100	245 [130-557.5]	250 [135-472.5]	0.250	-	-	-

Baseline IgE (IU/Ml)	96	402 [169-644]	297 [134.5-750]	0.786	-		
Years with omalizumab	110	2.5 [1-3.25]	2.5 [1-5]	0.793	-		
Omalizumab dose (mg/4 weeks)	110	575 [300-600]	300 [300-600]	0.218	-	-	-

BMI, body mass index; GERD, gastro-oesophageal reflux disease; SAHS, sleep apnoea-hypopnoea syndrome; COPD, chronic obstructive pulmonary disease; EC, eosinophilic asthma; ICS, inhaled corticosteroids; OCS, oral corticosteroids; FEV1, peak expiratory volume in the first second of forced expiration; ACT, Asthma Control Test; IgE, immunoglobulin E; OR, Odds ratio; CI95%, 95% confidence interval.

*Fisher's exact test
